# ASSOCIATION OF DIABETES WITH BODY COMPOSITION, CARDIORESPIRATORY FITNESS, AND MITOCHONDRIAL ENERGETICS

**DOI:** 10.1093/geroni/igad104.2085

**Published:** 2023-12-21

**Authors:** Sofhia Ramos, Giovanna Distefano, Philip Kramer, Peggy Cawthon, Frederico Toledo, Steven Cummings, Bret Goodpaster, Paul Coen

**Affiliations:** AdventHealth, Orlando, Florida, United States; AdventHealth, Orlando, Florida, United States; Wake Forest University School of Medicine, Winston-Salem, North Carolina, United States; California Pacific Medical Center, Research Institute, San Francisco, California, United States; University of Pittsburgh, School of Medicine, Pittsburgh, Pennsylvania, United States; California Pacific Medical Center Research Institute, San Francisco, California, United States; AdventHealth, Orlando, Florida, United States; AdventHealth Research Institute, Orlando, Florida, United States

## Abstract

We investigated the association of diabetes with cardiorespiratory fitness and skeletal muscle mitochondrial energetics in older adults (N=876, mean age ± SD: 76.3 ± 5.0 yrs.; 59% females) from the Study of Muscle, Mobility and Aging (SOMMA). Participants were grouped by self-reported diabetes status (N=131, with diabetes). Cardiorespiratory fitness (modified Balke protocol), mitochondrial energetics (31P magnetic resonance spectroscopy [31P-MRS] and high resolution respirometry from skeletal muscle biopsy), physical activity (wearable accelerometer) and body composition (magnetic resonance imaging) were measured. Generalized linear regression models were used to calculate means adjusted by age, race, gender, BMI, and co-morbidities. Thigh fat-free muscle volume (p=0.12) and abdominal subcutaneous adipose tissue depot (p=0.15) were similar between those with and without diabetes. However, visceral (18.84%, p>0.01) and intramuscular adipose tissue (6.57%, p=0.02) were higher in older adults with diabetes. VO2peak (-4.79%, p>0.01), and mitochondria energetics measured by ATPmax (-5.10%, p=0.04), maximum complex I+II carbohydrate (maxOXPHOSCHO) (-6.67%, p=0.02) and fatty acid (maxOXPHOSFAO) (-8.70%, p=0.01) stimulated respiration were lower in those with diabetes compared to those without. Following further adjustments for both physical activity and visceral adiposity, revealed that only VO2peak (-4.65%, p>0.01) and maxOXPHOSFAO (-7.38%, p=0.02) remained significantly lower in older adults with diabetes. Overall, our data suggests that cardiorespiratory fitness and skeletal muscle mitochondria energetics are reduced in older adults with diabetes, independent of visceral adiposity and physical activity.

